# PAK4 suppresses PDZ-RhoGEF activity to drive invadopodia maturation in melanoma cells

**DOI:** 10.18632/oncotarget.12282

**Published:** 2016-09-27

**Authors:** Nicole S. Nicholas, Aikaterini Pipili, Michaela S. Lesjak, Simon M. Ameer, Jenny L. C. Geh, Ciaran Healy, Alistair D. MacKenzie Ross, Maddy Parsons, Frank O. Nestle, Katie E. Lacy, Claire M. Wells

**Affiliations:** ^1^ Division of Cancer Studies, New Hunts House, Guy's Campus, King's College London, London, UK; ^2^ National Institute for Health Research (NIHR) Biomedical Research Centre, Guy's and St Thomas's Hospital and King's College London, London, UK; ^3^ Department of Plastic and Reconstructive Surgery, Guy's and St Thomas' Hospital, London, UK; ^4^ Randall Division, New Hunts House, Guy's Campus, King's College London, London, UK; ^5^ St Johns Institute of Dermatology, Guy's Hospital, London, UK

**Keywords:** PAK, melanoma, invadopodia, RhoA, PDZ-RhoGEF

## Abstract

Cancer cells are thought to use actin rich invadopodia to facilitate matrix degradation. Formation and maturation of invadopodia requires the co-ordained activity of Rho-GTPases, however the molecular mechanisms that underlie the invadopodia lifecycle are not fully elucidated. Previous work has suggested a formation and disassembly role for Rho family effector p-21 activated kinase 1 (PAK1) however, related family member PAK4 has not been explored. Systematic analysis of isoform specific depletion using *in vitro* and *in vivo* invasion assays revealed there are differential invadopodia-associated functions. We consolidated a role for PAK1 in the invadopodia formation phase and identified PAK4 as a novel invadopodia protein that is required for successful maturation. Furthermore, we find that PAK4 (but not PAK1) mediates invadopodia maturation likely via inhibition of PDZ-RhoGEF. Our work points to an essential role for both PAKs during melanoma invasion but provides a significant advance in our understanding of differential PAK function.

## INTRODUCTION

Whilst the survival rate for patients suffering from early stage melanoma is good, a significant proportion of patients will go on to develop metastatic disease [1]. Metastasis requires the coordinated rearrangement of the actin cytoskeleton as the melanoma cells move through tissue, a process thought to be regulated by Rho Family GTPases and their downstream effectors [2]. Moreover, during stromal invasion cancer cells are believed to form protease secreting invasive protrusions rich in actin [3, 4] termed invadopodia. Invadopodia are now reported both *in vitro* and *in vivo* and recent evidence suggests the invasive protrusion plays an active role in promoting metastasis [5–7].

The p-21 activated kinase (PAK) family of serine/threonine kinases are known effectors of Rho GTPases that control cytoskeletal dynamics and cell movement [2]. Human PAKs consist of 6 isoforms, which are separated into two groups according to their sequence and structural homology: group I, containing PAKs 1-3; and group II, containing PAKs 4-6. The overexpression of PAKs is found in a wide variety of human cancers and is often associated with an increase in invasive potential [2]. Indeed, PAK1 has been shown to localise to invadopodia protrusions [8], however, studies investigating the specific function of this protein in invadopodia formation/function have yielded conflicting results. To date, no studies have suggested a role for PAK4 in the invadopodia lifecycle. Moreover, the protein expression level and functional properties of the PAKs in melanoma invasion has not been explored.

PAK1 and PAK4 exhibit less than 55% sequence homology suggesting that these family members could drive divergent functions [9]. However, whilst multiple common substrates have been identified (e.g LIMK [10, 11], paxillin [12, 13]) there are virtually no confirmed isoform specific substrates reported [2] and directly comparable functional studies of PAK1 and PAK4 are rare. There is a particular level of complexity surrounding the role of PAK1/PAK4 in regulation of RhoA activity. PAK4 is purported to contain a GEF interacting domain (GID) [14] not found in PAK1, however both PAK1 and PAK4 have been reported to inhibit RhoA activator, GEF-H1 [14–16]. Nevertheless, whilst it has been previously reported that PAK4 depletion can elevate the level of RhoA activity [17], in contrast RhoA activation has not been observed in PAK1 depleted cells [18]. Interestingly, PAK1 and PAK4 may exhibit differential binding to a second RhoA activator, PDZ-RhoGEF [19, 20] a protein recently associated with invadopodia [21]. However, to date the PAK4:PDZ-RhoGEF interaction has not been associated with cellular activity. Despite the difficulties in separating PAK1 and PAK4 function mouse knockout (KO) phenotypes suggest that at least for PAK4 there are isoform specific functions as PAK4 KO mice are embryonically lethal whilst PAK1 KO mice remain viable and fertile [22, 23].

In this study we demonstrate that PAK1 and PAK4 expression at the protein level is significantly increased in melanoma compared to melanocyte controls using both cell lines and patient derived cell strains. Moreover we find a correlation between invasive potential and PAK expression. Our subsequent systematic analysis of isoform specific depletion in invasive cells has revealed that PAK1 and PAK4 are both required for *in vitro* and *in vivo* invasion. In addition our approach has allowed us to detect isoform specific functions during the invadopodia life cycle whereby PAK1 functions early in formation whilst PAK4 drives maturation. We have been able to demonstrate that PAK4, and not PAK1, regulates the activity levels of RhoA in invasive cells. Furthermore we find that during invadopodia maturation PAK4 is required to suppress RhoA activity in the invadopodia via inhibition of PDZ-RhoGEF. Taken together our work points to essential requirements for both PAK1 and PAK4 during melanoma invasion and further provides clear evidence of differential function.

## RESULTS

### PAK1 and PAK4 expression correlates with invasive potential

**Figure 1:**
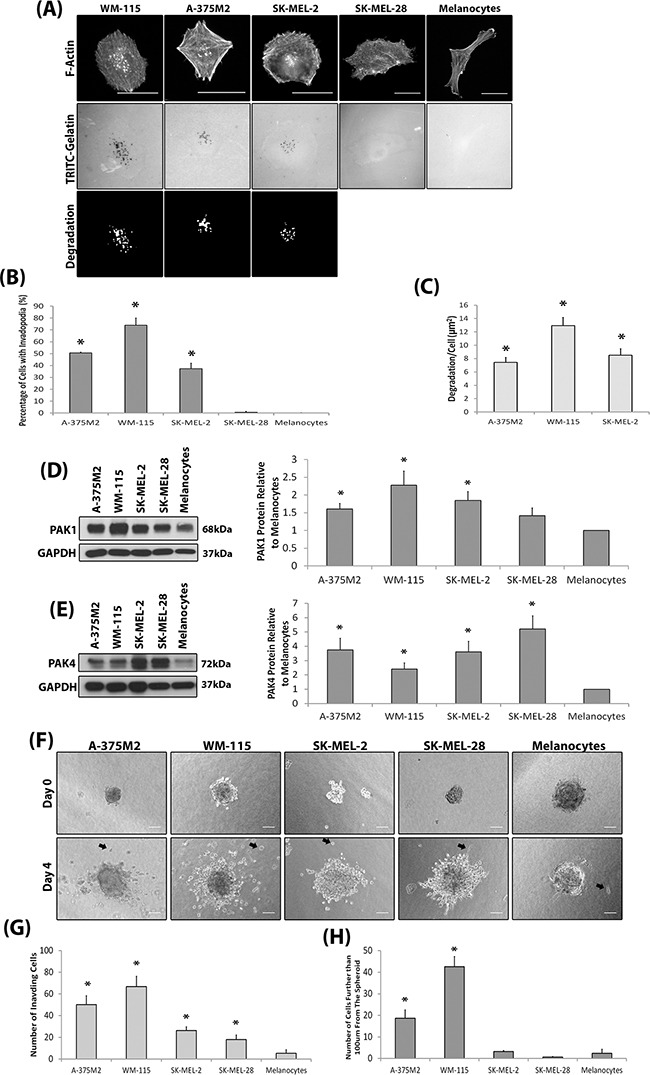
Invasive melanoma cell lines overexpress PAK **A.** Representative invadopodia assay images. Cells were seeded on rhodamine conjugated gelatin for 3 hrs and stained for F-actin. Actin rich dots corresponding with gelatin degraded dots were counted as invadopodia. The degradation was measured using ImageJ software. Scale bars = 20μm. **B.** The percentage of cells with invadopodia. Significance was calculated to a melanocyte control. 150 cells, over 3 independent experiments; * = P 00< 0.05. **C.** The area of degradation from invadopodia per cell. Significance was calculated between all cell lines. 90 invadopodia producing cells, over 3 independent experiments; * = P 00< 0.05. **D-E.** Western blot of PAK1 (D) and PAK4 (E) expression in melanoma cell lines compared to melanocytes. over 3 independent experiments; *= P 00< 0.05. Densitometric data were normalized to a GAPDH loading control. **F.** Spheroid assay. Examples of invading cells are indicated by black arrows. Scale bar = 100μm. **G.** Quantification of the number of cells that had invaded surrounding matrix **H.** the number of cells that invaded further than 100μm from the spheroid mass. Cells were seeded as determined by a proliferation assay (Supplementary Figure S1B). Significance was calculated to melanocytes. of 9 spheroids over 3 independent experiments; * = P 00< 0.05. In all cases data are mean values ± S.E.M.

**Figure 2:**
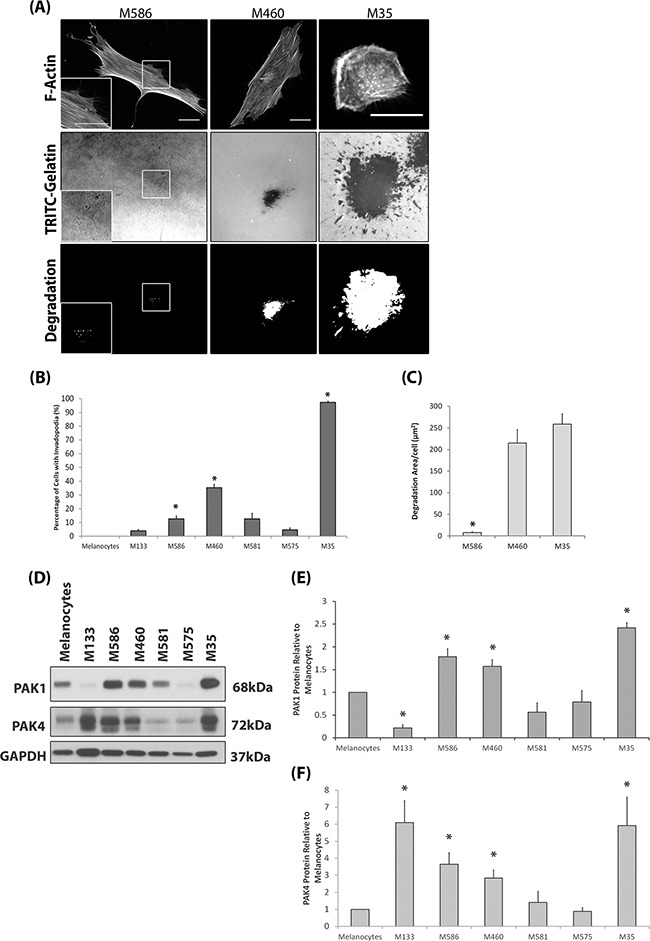
Invasive Patient Derived Cell Strains Overexpress PAK1 and PAK4 **A.** Representative images of the patient derived cell strain invadopodia assay. Patient derived cell strains were derived from primary (M133, M586 and M460) or metastatic tissue (M581, M575 and M35). Magnification box present in bottom left corner of M586 images, indicated by white borders. Cells were seeded on rhodamine conjugated gelatin for 24hrs and stained for F-actin. Invadopodia were scored as described in Figure 1. The degradation was measured using ImageJ software. Scale bars = 20μm. **B.** The percentage of cells with invadopodia. Significance was calculated to melanocytes. of 150 cells, over 3 independent experiments; * = P 00< 0.05. **C.** The area of degradation per cell. Significance was calculated between all cell lines. 90 invadopodia producing cells, over 3 independent experiments; * = P 00< 0.05. **D-F.** Western blot of PAK1 and PAK4 protein expression in patient derived cell strains and melanocytes. Significance was calculated to melanocytes (1). 3 independent experiments; *= P 00< 0.05. Densitometric data were normalized to a GAPDH loading control. In all cases data are mean values ± S.E.M.

We sought to initially define the invasive potential of a panel of melanoma cell lines and subsequently correlate invasive potential with PAK expression levels. We have adopted the invadopodia assay [24–26] and 3D spheroid assay [27–29] as our measures of invasive potential. Melanocytes do not produce invadopodia and not all melanoma derived cells lines are able to produce invadopodia (Figure 1A–1C); validated by the co-localisation of cortactin with F-actin staining and gelatin degradation (Supplementary Figure S1A) which defines completion of the invadopodia lifecycle. Interestingly, both PAK1 and PAK4 were overexpressed in those cell lines which were able to form invadopodia (Figure 1D–1E). Importantly, ability to form invadopodia was semi-predictive of 3D invasion potential (Figure 1F–1H) when cell proliferation rates were normalised (Supplementary Figure S1B). Where the two cell lines with the highest level of invadopodia activity (Figure 1B) also achieved the highest level of cell invasion (Figure 1H). Of the other PAK isoforms, only PAK2 was found to be overexpressed in melanoma cell lines, compared to melanocytes (Supplementary Figure S1C). This is not surprising given that PAK2 expression is already known to be overexpressed in melanoma cells [30] and important in invasion [31]. Recent publications have suggested that the formation of invadopodia is not restricted to cell culture conditions [5, 6], however, human cells directly derived from melanoma patients have not been tested. Interestingly, we found that not all patient cell strains were able to exhibit invadopodia activity (Figure 2A–2C) and invadopodia formation was not always correlated with pathological classification at time of biopsy in terms of origin; primary versus metastatic lesion (Table 1). However, we consistently detected high levels of PAK1 and PAK4 expression in the invasive cell strains, particularly M35 (Figure 2D–2F). Moreover, PAK1 and PAK4 were expressed in all the patient samples tested.

**Table 1:** Clinical data for patient derived cell strains

Cell Strain	Origin	Patient Stage at Time of Biopsy	Age at Time of Biopsy	Disease Progression	BRAF Mutation (V600E)
M35	Metastasis	IIIC	57	YES	Mutated
M460	Primary	IIIA	44	YES	Wildtype
M575	Metastasis	IIIB	92	YES	Unknown
M586	Primary	IIC	92	NO	Unknown
M581	Metastasis	IIIB	71	NO	Wildtype
M133	Primary	IIC	75	NO	Unknown

Patient staging according to the AJCC staging system [71]. Disease progression indicated an increase in staging or patient death from melanoma between the date of the biopsy excision and August 2014

### PAK1 and PAK4 are required for melanoma cell invasion *in vitro* and *in vivo*

**Figure 3:**
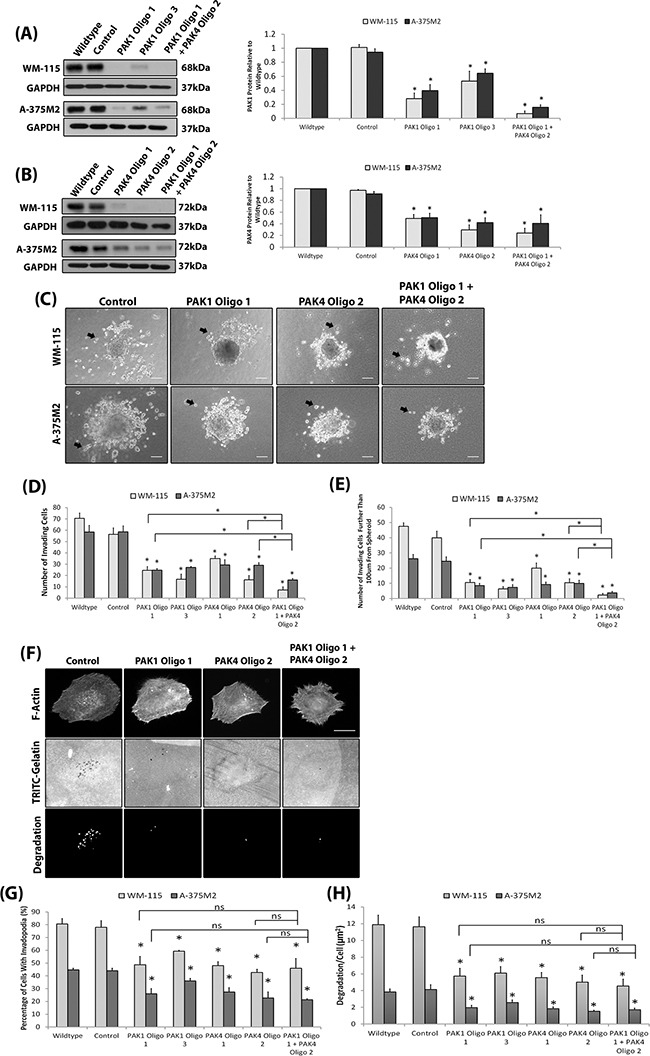
Depletion of PAK protein expression reduces invadopodia formation **A-B.** Transient depletion of PAK1 (A) and PAK4 (B) expression in the WM-115 and A-375M2 cell lines at 4 days post siRNA transfection. A double knockdown was performed using PAK1 Oligo 1 and PAK4 Oligo 2 oligonucleotides. Control cells were transfected with a control non targeting siRNA. Significance was calculated for protein depleted cell lines compared to wildtype. over 3 independent experiments; *= P 00< 0.05. Densitometric data were normalized to GAPDH, which was used as a loading control. **C.** Representative images of the 3D spheroid invasion assay Examples of invading cells are indicated by black arrows. Scale bar = 100μm. **D.** Quantification of the number of cells that had invaded surrounding matrix **E.** the number of cells that invaded further than 100μm from the spheroid mass. Significance was calculated to wildtype. 9 spheroids over 3 independent experiments; * = P 00< 0.05. **F.** Representative invadopodia assay images of WM-115 cells in which PAK1 and PAK4 proteins are depleted. The degradation was measured using ImageJ software. Scale bars = 10μm. The percentage of cells with invadopodia **G.** and the area of degradation from invadopodia per cell **H.**. Significance was calculated to wildtype cells. 150 (Percentage) or 90 (Degradation) cells, over 3 independent experiments; * = P 00< 0.05. In all cases data are the mean values ± S.E.M.

**Figure 4:**
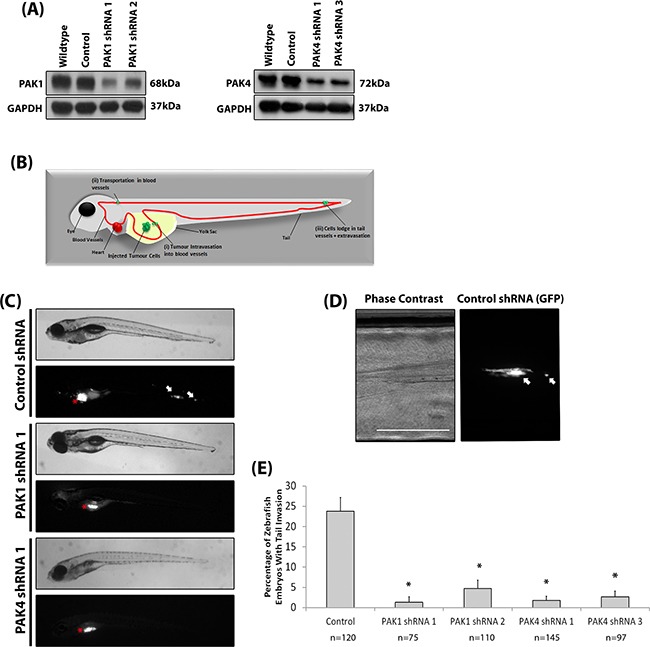
Depletion of PAK reduces melanoma invasion in vivo **A.** Stable reduction of PAK1 and PAK4 in the A-375M2 cell line. **B.** Diagrammatic representation of the invasive cell movement during the yolk invasion assay, including: (i) Tumour intravasation into blood vessels; (ii) Transportation in the blood vessels; and (iii) cells lodge in tail vessels and extravasate. **C.** Phase contrast and fluorescent images of zebrafish embryos at 4 dpi for embryos injected with control, PAK1 and PAK4 depleted cells (C). Red stars indicate cell mass in yolk sac (original injection site). White arrows indicate tail invasion of GFP labelled cells. Scale bar = 500μm. **D.** High magnification of tail invasion **E.** The percentage of embryos with cell tail invasion. Significance was calculated compared to control shRNA cells. Data are the mean values ± S.E.M., over at least 3 independent experiments; n = total number of embryos, *= P 00< 0.05.

Our results suggest that both PAK1 and PAK4 could play a role during melanoma invasion. To further investigate specific PAK1 and PAK4 functionally the two proteins were depleted in two invasive melanoma cell lines (WM-115 and A-375M2) (Figure 3A–3B). We were able to sustain siRNA induced PAK1 and PAK4 depletion for seven days (Supplementary Figure S1D-S1E). Reduced expression of PAK1 and PAK4 in both WM-115 and A-375M2 cells decreased invasion (Figure 3C) and double depletion of PAK1 and PAK4 expression resulted in a further reduction in invasive potential (Figure 3C–3E). Depletion of PAK1 and PAK4 also decreased the percentage of cells that produced degradative invadopodia in both WM-115 and A-375M2 cells (Figure 3F–3H). Moreover, treatment of cells with the PAK1 specific inhibitor IPA-3 significantly reduced the level of invadopodia formation (Supplementary Figure S1F) confirming the dependence on PAK1 kinase activity previously reported [26]. Having established a requirement for PAK expression *in vitro* we then sought to translate our findings *in vivo*. We generated stable shRNAi bi-cistronic GFP control, PAK1 or PAK4 depleted A-375M2 cell lines [17] (Figure 4A) and performed the *in vivo* the zebrafish yolk invasion assay [32–34] (Figure 4B). Cells were injected into the yolk sac of zebrafish embryos at 2 days post fertilisation (dpf). Control cells were able to invade through the yolk and intravasate into the embryo vasculature where they lodged in the tail (Figure 4B–4E). However, tail invasion events were significantly reduced using cells with stably depleted PAK1 or PAK4 expression (Figure 4C–4E). Therefore, using three different experimental assays we have demonstrated that reducing PAK1 or PAK4 expression can inhibit the invasion of melanoma cells.

### PAK1 and PAK4 have differential functions in the invadopodia lifecycle

**Figure 5:**
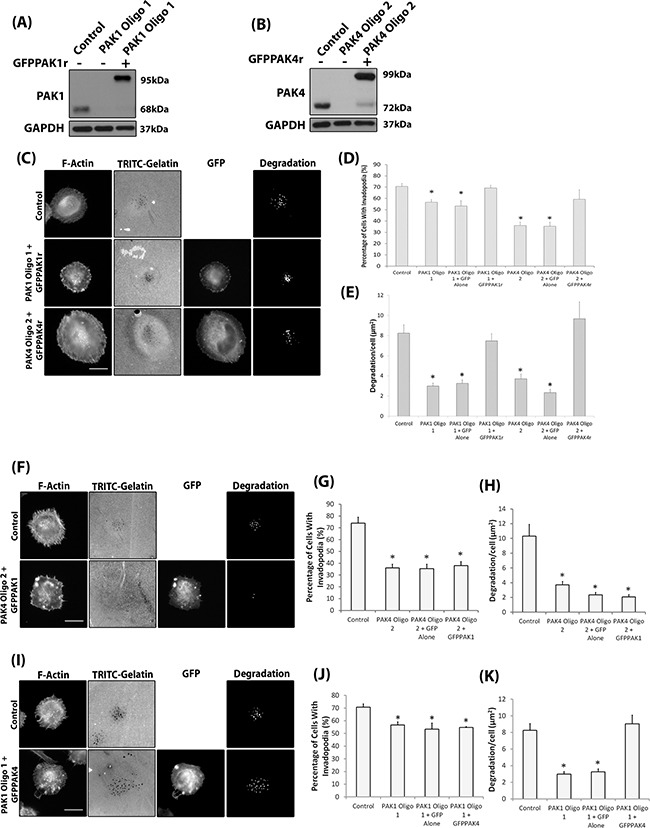
Isoform specific PAK1 and PAK4 functions during the invadopodia lifecycle **A-B.** Confirmation of siRNA resistant protein expression in PAK1 (A) or PAK4 (B) siRNA depleted WM-115 cells. **C.** Representative invadopodia assay images of WM-115 depleted cells transfected with GFPPAK1r or GFPPAK4r. Scale bars = 10μm. **D.** The percentage of cells with invadopodia and **E.** the area of degradation from invadopodia. Significance was calculated to control siRNA transfected cells of 150 (Percentage) or 90 (Degradation) cells, over 3 independent experiments; * = P 00< 0.05. **F.** Representative invadopodia assay images of WM-115 PAK4 knockdown cells transfected with GFP alone or GFPPAK1. Scale bars = 10μm. **G.** The percentage of cells with invadopodia. Significance was calculated to control siRNA transfected cells. 150 cells, over 3 independent experiments; * = P 00< 0.05. **H.** The area of degradation from invadopodia per cell. Significance was calculated to control siRNA transfected cells. 90 invadopodia producing cells, over 3 independent experiments; * = P 00< 0.05. Control = cells transfected with non-specific siRNA. **I.** Representative invadopodia assay images of WM-115 PAK1 knockdown cells transfected with GFP alone or GFPPAK4. Scale bars = 10μm. **J.** The percentage of cells with invadopodia. Significance was calculated to control siRNA transfected cells. 150 cells, over 3 independent experiments; * = P 00< 0.05. **K.** The area of degradation from invadopodia per cell. Significance was calculated to control siRNA transfected cells. 90 invadopodia producing cells, over 3 independent experiments; * = P 00< 0.05. Control = cells transfected with non-specific siRNA. In all cases data are mean values ± S.E.M

**Figure 6:**
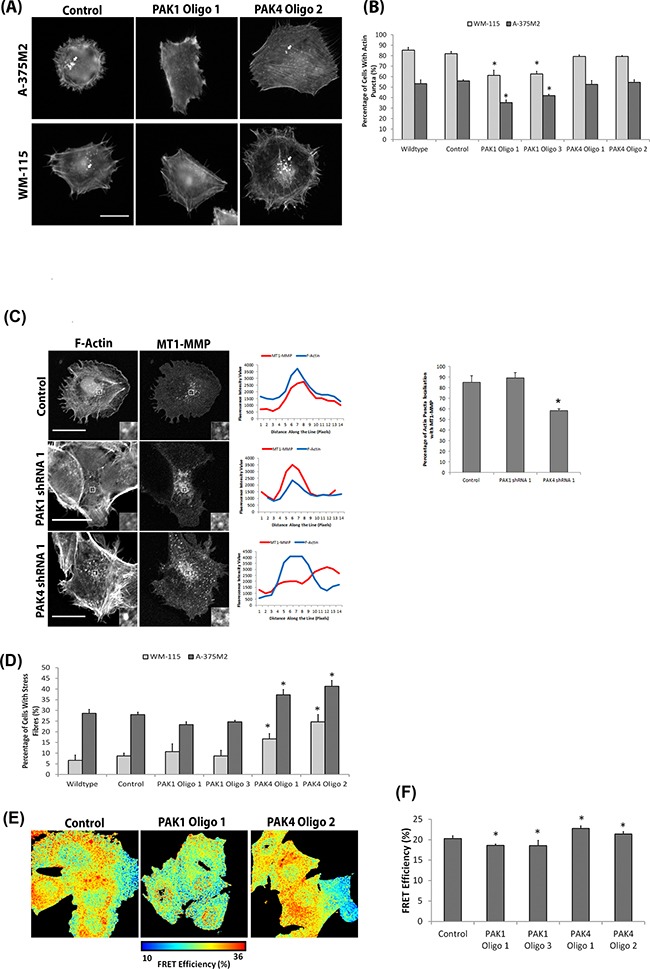
Differential PAK1 and PAK4 signalling in invasive cells **A.** Representative F-actin images of WM-115 and A-375M2 cells with deleted PAK1 and PAK4 expression (individually and simultaneously). Scale bar = 10μm. **B.** The percentage of cells with actin puncta on gelatin. Significance was calculated to wildtype cells. 150 cells, over 3 independent experiments; * = P 00< 0.05. **C.** Co-localisation of mCherry-MT1-MMP with F-actin on gelatin. Scale bar = 20μm. The percentage of cells with actin puncta localisation with MT1-MMP. Significance was calculated to wildtype cells. 30 cells, over 3 independent experiments; * = P 00< 0.05. **D.** Percentage of WM-115 and A-375M2 cells with prominent actin fibres on gelatin. Significance was calculated to wildtype cells. 150 cells, over 3 independent experiments; * = P 00< 0.05. **E-F.** FRET analysis of RhoA activation in A-375M2 RhoA cells in which PAK1 and PAK4 expression was diminished. Significance was calculated to control cells over 3 independent experiments; * = P 00< 0.05. In all cases data are mean values ± S.E.M.

Whilst our work has outlined important roles in melanoma invasion we have not identified any differential function. Initially, we validated a specific requirement for PAK1 and PAK4 isoforms by re-expressing siRNA resistant GFPPAK1r and GFPPAK4r (Figure 5A–5B) in a depleted background. Under these conditions invadopodia formation and degradation were restored back to control levels (Figure 5C–5E). We then proceeded to test PAK isoform redundancy. Expression of PAK1GFP in PAK4 depleted cells was unable to recover the percentage of cells with invadopodia, nor the level of matrix degradation (Figure 5F–5H), thus increased levels of PAK1 are unable to compensate for loss of PAK4. Exogenous expression of PAK4GFP, in contrast, whilst unable to elevate the percentage of cells with invadopodia, did induce an increase in maturation in those invadopodia present in a PAK1 depleted population (Figure 5I-5K). This suggests that PAK1 and PAK4 have unique functions in invadopodia dynamics and that PAK4 may act downstream of PAK1. Having identified a potential differential function we more carefully examined the presence of actin puncta as an indicator of invadopodia initiation. We noted that whilst depletion of PAK1 expression reduced the percentage of cells with actin puncta (initiation of invadopodia formation [35]), reduction of PAK4 expression had no effect on the percentage of cells with actin puncta (Figure 6A–6B).

### PAK4 depleted cells fail to efficiently complete the invadopodia lifecycle

Our data (Figure 5 & 6) suggest that although the outcome of both PAK1 and PAK4 expression is a loss of degradation, PAK1 is required in the formation stage, whilst PAK4 activity is required during the later maturation stage. To further explore the failure of PAK4KD cells to complete the lifecycle we monitored the localisation of MT1-MMP to the actin puncta. We observed a significant reduction in MT1-MMP localisation to nascent invadopodia in our PAK4 depleted cells (Figure 6C). Thus these data further support differences in the functional role of PAK1 and PAK4 during the invadopodia lifecycle.

### PAK4 signals via PDZ-RhoGEF to promote invadopodia function

**Figure 7:**
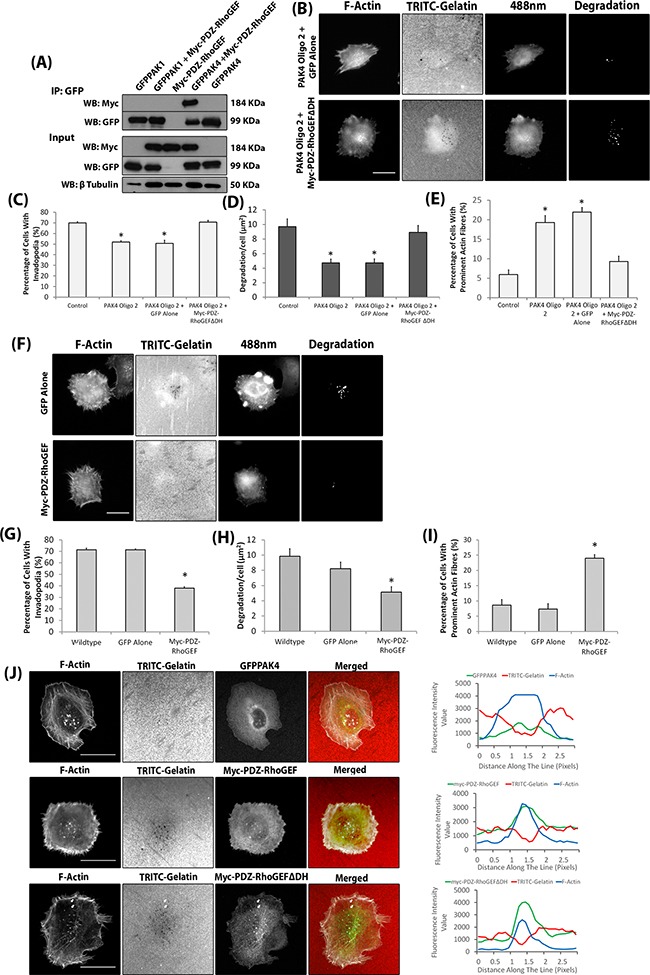
PAK4 mediates invadopodia dynamics via PDZ-RhoGEF **A.** Immunoprecipitation in HEK293 cells of GFP-PAK1/PAK4 in the presence/absence of myc-PDZ-RhoGEF expression with a myc-PDZ-RhoGEF alone control. The Immunoprecipitation was probed for presence of myc-PDZ-RhoGEF **B.** Representative images of PAK4 knockdown WM-115 cells expressing myc-PDZ-RhoGEFΔDH. Scale bars = 10μm. The percentage of cells with invadopodia **C.** the area of degradation **D.** and the percentage of cells with prominent actin fibres **E.** in PAK4 knockdown cells expressing myc-PDZ-RhoGEFΔDH. Significance was calculated to control WM-115 cells. Data are mean values ± S.E.M. of 150 (Percentage of invadopodia and prominent actin fibres) or 90 (Degradation) cells, over 3 independent experiments; * = P 00< 0.05. **F.** Representative images of WM-115 cells expressing myc-PDZ-RhoGEF. Scale bars = 10μm. The percentage of cells with invadopodia **G.** the area of degradation **H.** and the percentage of cells with prominent actin fibres **I.** in cells expressing myc-PDZ-RhoGEF. Significance was calculated to wildtype WM-115 cells. **J.** Co-localisation of GFPPAK4 and myc-PDZ-RhoGEF (wildtype and ΔDH mutant) with F-actin and TRITC gelatin degradation. Scale bar = 10μm.

Having identified specific roles for PAK1 and PAK4 during the invadopodia lifecycle we proceeded to focus on identifying an underlying molecular mechanism. PAK1 has already been reported to drive invadopodia formation via phosphorylation of cortactin and thus we focussed on PAK4, which had not previously been associated with invadopodia activity. We noted that PAK4 but not PAK1 depleted cells exhibited a significantly increased level of actin stress fibres (Figure 6D). An increase in prominent actin fibres has been linked to cell rigidity and reduced cell invasion [36]. Indeed, an increase in prominent actin fibres was reported in PAK4 depleted DU-145 cells [17]. Here, PAK4 depletion induced stress fibre formation was attributed to an increased level of GTP-loaded RhoA [17]. We have used a RhoA biosensor [37] to quantitatively measure the level of RhoA activation in individual cells. Increased RhoA activity was detected in cells when PAK4 levels were reduced, compared to the control cells (Figure 6E–6F). Conversely, when PAK1 levels were diminished there was a decrease in detectable RhoA activity, compared to the control (Figure 6E–6F). Thus, the level of RhoA activation differs in PAK1 and PAK4 knockdown cells. We have shown that specific loss of PAK4 expression concomitantly reduces the invasive potential of melanoma cells and increases the level of RhoA activity. Whilst previous work has indicated that reduced RhoA expression inhibits invadopodia formation [38] there are also reports that a constitutively active RhoA mutant reduced the podosome induced degradation by v-Src transformed NIH3T3 cells [39]. Together these findings suggest that a balance of RhoA activity and inactivation may be important for function. Therefore, we hypothesised that PAK4 may be required to reduce levels of RhoA activation during the invadopodia maturation stage. PAK4 has previously been shown to inhibit the activation of RhoA via the phosphorylation and inhibition of Rho guanine nucleotide exchange factors (GEFs) such as GEF-H1 [14, 17]. However, initial experiments found no modulation of GEF-H1 phosphorylation in cells with PAK1/PAK4 depletion (Supplementary Figure S2A-S2B). Indeed, interaction with GEF-H1 would not differentiate between PAK1 and PAK4 [16, 17, 40]. In contrast, previous reports have suggested that PAK4 (but not PAK1) can bind to, and inhibit the GEF activity of, PDZ-RhoGEF [19, 20]. Moreover, PDZ-RhoGEF has recently been reported to function in invadopodia^27^. We found PDZ-RhoGEF is expressed in both of our invasive melanoma cells (Supplementary Figure S2C) and we have now confirmed the preferential binding of PAK4 to PDZ-RhoGEF (Figure 7A). Thus PAK4 regulation of RhoA activity during the invadopodia life cycle could occur via PDZ-RhoGEF. To investigate our hypothesis, a dominant negative PDZ-RhoGEF mutant (myc-PDZ-RhoGEFΔDH [41]) was expressed in cells. This mutant is reported to inhibit RhoA activity [41, 42] and should therefore be able to rescue the PAK4 depletion phenotype. Expression of myc-PDZ-RhoGEFΔDH was able to rescue the percentage of cells with degradative invadopodia in PAK4 depleted cells to control levels (Figure 7B–7D) concomitant with a reduction in prominent actin fibres (Figure 7E). Moreover, importantly myc-PDZ-RhoGEFΔDH expression was unable to rescue the percentage of cells with invadopodia or the level of gelatin degradation in PAK1 depleted cells (Supplementary Figure S2D-2F). This further supports a specific PAK4:PDZ-RhoGEF interaction to inhibit RhoA activity during invadopodia maturation. Thus we predicted that an increase in RhoA activity by overexpression of PDZ-RhoGEF should negatively interfere with invadopodia maturation. In line with this hypothesis we find that exogenous expression of myc-PDZ-RhoGEF in WM-115 cells reduced invadopodia maturation compared to wildtype cells, mimicking the PAK4 knockdown phenotype (Figure 7F–7H). Moreover, PDZ-RhoGEF expression increased the percentage of cells with prominent actin fibres compared to wildtype cells (Figure 7I). Thus we predict a novel role for both PAK4 and PDZ-RhoGEF where PAK4 drives invadopodia maturation via inhibition of PDZ-RhoGEF induced RhoA activity, indeed a kinase dead variant of PAK4 cannot rescue the loss of invadopodia phenotype (Supplementary Figure S3). Interestingly, we are able to localise both GFPPAK4 and myc-PDZ-RhoGEF to invadopodia structures (Figure 7J), suggesting that PDZ-RhoGEF activity is required earlier in the invadopodia lifecycle but that inhibition by PAK4 is essential to achieve maturation.

## DISCUSSION

PAK1 and PAK4 protein levels were elevated in invasive melanoma cell lines and cells derived from patient samples. This study used the invadopodia assay as an indicator of invasive potential, alongside a 3D and an *in vivo* invasion assay, to investigate the role of PAK1 and PAK4 in melanoma invasion. Whilst PAK1 activity was clearly associated with nascent invadopodia formation PAK4 depletion revealed a differential role during invadopodia maturation. Thus by systematic analysis of PAK isoform deletion we have been able to assign differential function. Moreover, subsequent studies were able to specifically assign a PAK4:PDZ-RhoGEF interaction to the invadopodia maturation stage. These data therefore reveal novel functions for both PAK4 and PDZ-RhoGEF and provide clear evidence of differential function between these two widely studied by rarely compared PAK family members.

In both melanoma cell lines and patient derived cell strains a positive correlation was observed between the level of PAK1 protein expression and cell invasiveness. These findings complement previous studies linking PAK1 with increased invasiveness of uveal melanoma [43]. This suggests a global melanoma requirement for PAK1. Furthermore, PAK1 was found to be overexpressed in mouse malignant squamous cell carcinoma (SCC) [44]. Previous research suggested that PAK1 overexpression is restricted to a wildtype BRAF subset [45]. Though possibly the case for primary melanoma, our study has shown that this is not the case when investigating metastatic melanoma, where PAK1 overexpression correlates with invasion (rather than BRAF mutational status).

PAK4 was robustly overexpressed in the melanoma cell lines, including the invasive cells. However, high levels of PAK4 alone did not specifically correlate with invasive ability. Rather we would suggest that cells need high levels of both PAK1 and PAK4 to achieve efficient invasion. A hypothesis which fits with PAK1 being upstream of PAK4 in the invadopodia lifecycle. An increase in PAK4 mRNA in melanoma cell lines, compared to melanocytes, suggests that the upregulation may also be occurring at the transcriptional level [46]. PAK isoforms (of which there are 6) are overexpressed in a wide variety of human tumours such as breast, colon, prostate and ovarian cancer [47]. PAK1 has been previously found to promote the cell invasion of colon cancer [48] and breast cancer cell lines [18]. Similarly, PAK4 promotes the cell invasion of choriocarcinoma [49] and endometrial cancer [50]. Moreover, in skin cancer specifically, PAK1 and PAK4 promote cell invasion of uveal melanoma [43] and SCC [44] cell lines, respectively. However, the role of these proteins in skin melanoma invasion is undefined. We found that depleting either PAK1 or PAK4 significantly reduced invadopodia maturation and subsequent matrix degradation in melanoma cells and found that PAK1 or PAK4 expression are required for efficient invasion in 3D and *in vivo* invasion assays. The data presented here complements previously published studies with other cancer types and suggests a global involvement for PAK1 and PAK4 in cancer invasion at least *in vitro* [18, 43, 44, 48–50].

Previous studies have specifically shown that PAK1 localises to invadopodia [8] and that the inhibition of endogenous PAK1 via an autoinhibitory domain fragment, can reduce the formation of invadopodia in A375MM cells [26]. Our findings complement this work, providing further evidence that PAK1 is important in invadopodia formation in melanoma. In contrast, PAK1 has been associated with Rac mediated invadopodia dissolution in a breast cancer cell line, thus PAK1 function in invadopodia may be cell type specific [8]. PAK4 localises to and promotes the formation of podosomes, a structure that often shares similar proteins to invadopodia [51, 52]. However, the work presented here is the first to demonstrate that PAK4 is localised to invadopodia and involved in invadopodia maturation.

PAK1 and PAK4 are activated differently [2], however little is known about the unique signalling pathways or the substrates of these two family members and how these differences may impact on the effect that these proteins have on the invasive potential of tumours [23]. A better understanding of isoform specific differences in downstream signalling could help guide the further development of therapeutic agents. Currently, pharmaceutical companies are focused on developing group or isoform specific inhibitors [53–55]. Therefore, data indicating whether both groups contribute to invasion and metastasis in the same way, may determine whether the use of cross reactive inhibitors is more beneficial than isoform specific inhibitors in treating some cancers types. However, consideration of increased risk of side effects from a pan-PAK inhibitor would be critical.

Depletion of PAK1 reduced both the percentage of cells with invadopodia and also the matrix degradation for those cells that formed invadopodia. In addition, reduction of PAK1 protein expression also reduced the number of nascent invadopodia actin puncta, suggesting an important role for this protein early in the formation stage of the invadopodia lifecycle. Furthermore, PAK1 kinase activity was required for efficient invadopodia formation supporting a role for substrate phosphorylation in line with previous reports [26]. In contrast, PAK4 protein depletion did not reduce the formation of nascent actin puncta but did inhibit maturation. These findings are in agreement with previous studies that suggested PAK4 is required for podosome function [51, 52]. It is possible that PAK4 is responsible for promoting the expression of proteases involved in matrix degradation, as has been previously reported in other cell types, such as MMP-2 [50, 56, 57], MMP-9 and MT1-MMP [49]. However, we specifically localised PAK4 to mature invadopodia suggesting a structural/functional role at the protein level. Furthermore, we confirmed that PAK4, but not PAK1, can bind to PDZ-RhoGEF which was also localised to invadopodia. PDZ-RhoGEF is known to preferentially activate RhoA, over RhoB and RhoC [58], and previous work has demonstrated that PAK4 can negatively regulate PDZ-RhoGEF [19]. We now find that PAK4 depleted melanoma cells exhibit elevated levels of RhoA activity. Furthermore, PDZ-RhoGEF dominant negative mutants, while able to rescue invadopodia function in PAK4 depleted cells, have no effect on invadopodia formation in PAK1 depleted cells. The localisation of PDZ-RhoGEF within invadopdia^27^ suggests that there is a stage in the invadopodia life cycle when PDZ-RhoGEF mediated RhoA activity is required. Indeed, studies suggest that RhoA activity is essential during invadopodia formation [38] and to regulate MT1-MMP delivery [59]. However, work with podosomes suggests that a balance of RhoA activation and inactivation is important for podosome function [39, 60–62]. We detected a variation in the level of PDZ-RhoGEF expression between our invasive melanoma cell lines, however we would suggest that global expression levels are not necessarily an indicator of activity. Rather the importance is regulated spacial and temporal localisation within invadopodia. We hypothesise that RhoA inactivation is required to promote maturation; an inactivation delivered by the interaction between PAK4 and PDZ-RhoGEF. Indeed, expression of kinase dead PAK4 was unable to rescue the PAK4 depletion phenotype emphasising the need for kinase activity. We would speculate based on our MT1-MMP studies that this interaction is required for the final delivery of proteases involved in matrix degradation to the invadopodia core and/or retention of MMPs within the invadopodia structure; given that PAK4 and PDZ-RhoGEF are localised there. This hypothesis is supported by our observation that a dominant negative PDZ-RhoGEF can revert the loss of mature degradative invadopodia in PAK4 depleted cells. Our data clearly points to a specific role for PDZ-RhoGEF in regulating invadopodia dynamics however we cannot at this stage conclusively rule out the involvement of other RhoA regulators downstream of PAK4. Indeed, further examination of the relationship between PAK4 and PDZ-RhoGEF within the invadosome is warranted.

Little is known about the role that PDZ-RhoGEF plays in tumour invasion, although a potential role in invadopodia dynamics was recently reported [21]. Gene amplification of PDZ-RhoGEF is evident in gallbladder cancer specimens, compared to normal tissue [63] thus it will be of interest to observe if PDZ-RhoGEF levels are prognostic of invasive potential. Our hypothesis proposes that a reduction in RhoA activity is required to complete the invadopodia life cycle. This is consistent with a the recent finding that invadopodia activity requires suppression of Rho signalling [64] and previous work that suggests that efficient invasion requires localised suppression of Rho signalling [65–68]. Whilst our work highlights the importance of MT1-MMP delivery it could also be the case that a reduction in RhoA activity is required to reduce contractility and allow extension of the forming the protrusion [69] which would in turn promote MT1-MMP activity. It will be interesting to explore further the specific functional consequences of Rho suppression during the invadopodia lifecycle.

This study clearly demonstrates that PAK1 and PAK4 play an important role in melanoma cell invasion. In addition, we have been able to identify isoforms specific functions during the invadopodia life cycle; where PAK1 drives formation and PAK4 promotes maturation through the localised inhibition of PDZ-RhoGEF. Importantly, our data supports the development of pan-PAK inhibitors which block the function of both the group I and group II PAKs as a viable treatment option for metastatic melanoma.

## MATERIALS AND METHODS

### Antibodies and reagents

Anti-PAK1, anti-PAK2 and anti-GEFH-1 were purchased from Cell Signalling Technology. Anti-c-Myc and anti-PAK5 were acquired from Santa Cruz. Anti-Cortactin from Upstate. Anti-GAPDH from Millipore. Anti-p-GEFH-1 (Ser^885^) from Abcam. Anti-PAK3 from New England Biolabs and anti-PAK6 from Calbiochem. Anti-GFP from Roche Life Science. Anti-β-Actin and anti-β-Tubulin from Sigma Aldrich. Anti-HMWMAA and anti-human IgG kind gift from Sophia Karagiannis, King's College London (KCL). Anti-PAK4 was previously described [17]. Horseradish peroxidase (HRP) conjugated secondary antibodies were purchased from DAKO. The Alexa Fluor 488 conjugated antibodies and Phalloidin from Invitrogen. GFP-PAK1r, GFP-PAK4r, HA-PAK4r and HA-PAK4K350/351Mr were constructed by site-directed mutagenesis, according to the manufacturer's instructions, using the QuikChange Multisuite II kit (Stratagene). The Myc-PDZ-RhoGEF and Myc-PDZ-RhoGEFΔDH were kind gifts from John Masters, University College London (UCL). The RhoA Biosensor was generously provided by Maddy Parsons, (KCL). IPA-3 was purchased from Sigma.

### Cell culture

HEK 293 cells were grown in Dulbecco's modified eagle's medium (DMEM), the melanoma cell lines A-375M2, SK-MEL-2 and SK-MEL-28 were grown in Dulbecco's modified eagle's medium: nutrient F-12 ham (DMEM F-12) (containing L-glutamine), and the WM-115 cell line was grown in minimum essential medium (MEM) (containing L-glutamine). All the growth media supplemented with 10% foetal bovine serum (FBS), penicillin and streptomycin sulphate. Human melanocytes were cultured in epidermal melanocyte basal growth medium to which was added epidermal melanocyte growth supplement and antibiotic supplement, according to the manufacturer's instructions (TCS Cellworks). WM-115 cells were transiently transfected with X-tremeGENE HP transfection reagent and the A-375M2 cells using Lipofectamine® 2000 transfection reagent, according to the manufacturer's instructions. The human melanoma tumour tissues were obtained with written informed consent and all work was approved by the Guy's Research Ethics Committee, Guy's and St. Thomas' NHS Trust (reference number 08/H0804/139, approval date 15/10/2008). Isolation of melanoma cells was performed using fluorescence activated cell sorting (FACS) by flow cytometry. For inhibitor studies cells were incubated with DMSO or 5uM IPA-3 for 2 hours prior to the invadopodia assay.

### Si/Sh RNA

Oligonucleotides (Dharmacon, UK) were transiently transfected at a concentration of 25nM using HiPerFect transfection reagent (Qiagen), according to the manufacturer's instructions. ShRNAi vectors (pGIPz ;Open Biosystems), were transfected into cells using Lipofectamine® transfection reagent (Invitrogen). Knockdown cells were selected and maintained in media containing 1μg/ml puromycin.

### Western blotting

Proteins were separated by electrophoresis and transferred onto protran nitrocellulose hybridization transfer membranes. The membranes were blocked and subsequently incubated overnight at 4°C in primary antibody in TBST with 1% (w/v) non-fat milk powder or BSA. The membranes were washed three times in TBST and then incubated for 1 hr at room temperature with the respective HRP secondary in TBST with 1% (w/v) non-fat milk powder or BSA. Proteins were detected using Pierce® enhance chemiluminescence (ECL) western blotting substrate and analysed using ImageJ software.

### Immunofluorescence staining

Cells were fixed with 4% (w/v) paraformaldehyde (PFA) and permeabilised using 0.2% (v/v) triton X-100 and then washed with PBS. For FRET experiments, the autofluorescence was quenched in sodium borohydride. Non-specific binding was blocked by 3% BSA. Coverslips were incubated for 2 hours with the primary antibody and then washed with PBS. Cells were incubated for 1 hour with secondary antibody and fluorophore conjugated phalloidin. Coverslips were then washed with PBS and mounted using Fluorsave^™^ reagent or ProLong® Gold antifade reagent (FRET). Coverslips were visualised using either a Olympus Ix71 microscope or Nikon Eclipse Ti confocal microscope. Fluorescence intensity co-localisation was measured using ImageJ. FRET was measured using a multiphoton, time-correlated single-photon counting (TCSPC) fluorescence lifetime imaging microscope (FLIM). FRET efficiency was analysed using TRI2 software [70].

### Immunoprecipitation

Experiments were performed 48 hours post-transfection using GFP Trap® coupled to agarose beads (Chromotek) according to the manufacturer's protocol. Briefly, cells were washed 3 times with PBS and lysed in lysis buffer (10 mM Tris/Cl pH 7.5; 150 mM NaCl; 0.5 mM EDTA; 0.5% NP40). GFP Trap® beads were equilibrated in dilution/wash buffer (10 mM Tris/Cl pH 7.5; 150 mM NaCl; 0.5 mM EDTA) before being added to the cell lysates. Lysates were then incubated for 1 hour at 4°C with constant rolling. The centrifuged beads were washed 3 times with dilution/wash buffer and resuspended in 6x gel sample buffer.

### Invadopodia Assay

Briefly, rhodamine conjugated gelatin was prepared as previously described [25]. Ethanol washed coverslips were coated with rhodamine conjugated gelatin and fixed with gluteraldehyde. The fluorescence was quenched with sodium borohydride and washed three times with PBS. Cells were seeded on the gelatin coated coverslips and incubated for 3 hours at 37°C before being immunofluorescently stained. Gelatin degradation of each invadopodia producing cell was measured using the gelatin degradation plug-in with ImageJ software (a kind gift from Laura Machesky, Beatson Institute for Cancer Research, Glasgow).

### 3D Spheroid Invasion Assay

Spheroids were formed by incubating cells at 37°C for 3 days in media containing methylcellulose in 96-well U-bottomed suspension culture plates. Following this, the spheroids were transferred into collagen I matrix. Images were taken at day 0 and day 3 or 4 using an Olympus Ix71 microscope with Image-Pro Plus 7.0 software.

### MTT Assay

Cells were plated in a 96 well plate and left to grow for 4 days. Cells were stained with methylthiazolyldiphenyl-tetrazolium bromide (MTT) according to the manufacterers instructions and the absorbance at 570nm was measured using an Alpha-Fusion plate reader.

### Zebrafish Yolk Invasion Assay

All work that was conducted using zebrafish were performed under the UK Home Office project licence PPL 70/7912 and approved by the King's College Ethical Review committee. 2 dpf embryos were submerged in 3.5mM MS222, containing penicillin and streptomycin, and ~500 A-375M2 cells were injected into the embryo yolk sac using a Nikon SMZ-U zoom 1:10 Picospritzer II microinjection station. Injected embryos were placed in E3 media (containing penicillin and streptomycin) and incubated at 28°C for 1 hour to recover, then transferred to 35°C for the remainder of the experiment. 4 hours post-injection, embryos that lacked a clear tumour mass within the yolk sac or that had cells outside of the yolk sac were removed and humanely killed using 15mM MS222. The percentage of embryos with A-375M2 cell tail invasion was calculated 4 days post-injection. The embryos were then humanely killed by the addition of 15mM MS222 for 1 hour.

### Statistical analyses

Data sets were compared using two-tailed Students' t-tests and presented as mean ± SEM. Statistical significance was accepted for p ≤ 0.05.

## SUPPLEMENTARY FIGURES


