# Supplementary material for: PAK4 suppresses PDZ-RhoGEF activity to drive invadopodia maturation in melanoma cells
Source: Oncotarget. 2016 Sep 27;7(43):70881–97. doi: 10.18632/oncotarget.12282 (PMC5342596; doi:10.18632/oncotarget.12282)
Supplement: Supplementary file 1 [file oncotarget-07-70881-s001.pdf]

PAK4 suppresses PDZ-RhoGEF activity to drive invadopodia maturation in melanoma cells

SUPPLEMENTARY FIGURES

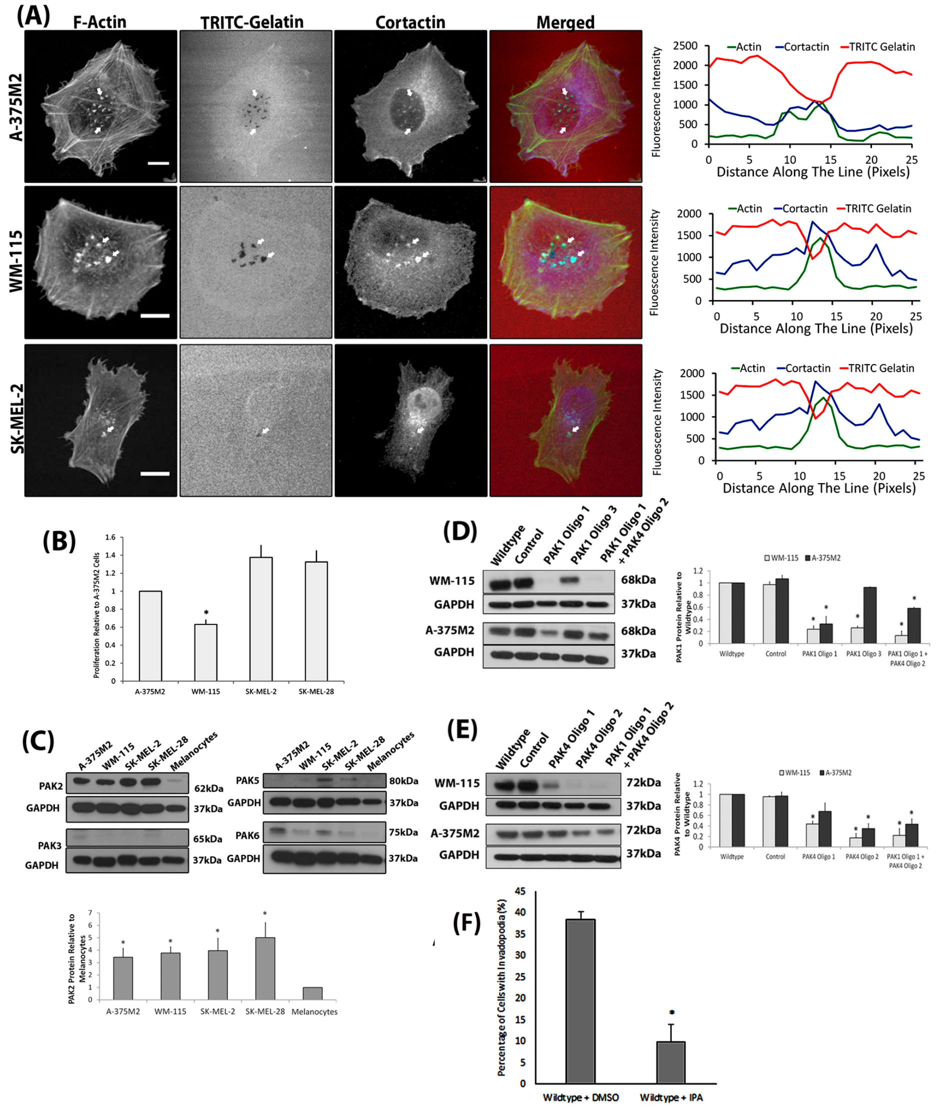

Supplementary Figure S1: Invasive phenotype of melanoma cells and PAK isoform expression. **A.** A-375M2, WM-115 and SK-MEL-2 cells were seeded on TRITC gelatin coated coverslips for 3 hrs and then fixed and stained for F-actin and cortactin. Scale Bar = 10um. Fluorescence intensity plots show co-localisation of cortactin, f-actin and gelatin degradation. **B.** MTT assay of melanoma cell lines. Cells were grown over 4 days. Following this, cells were incubated in MTT solution, solubilised in DMSO and absorbance was measured using a plate reader and normalised relative to A-375M2 cells. Significance was calculated between all cell lines. Data are mean values +/- S.E.M., over 3 independent experiments; *=P<0.05. **C.** Western blot of PAK2, PAK3,PAK5 and PAK6 protein expression in melanoma cell lines compared to melanocytes. Data are mean values +/- S.E.M., over 3 independent experiments; *=P<0.05. Densitometric data was quantified for PAK2 and was normalised to GAPDH as a loading control. PAK1 **D.** and PAK4 **E.** protein depletion was sustained for seven days from siRNA oligonucleotide transfection of WM-115 and A-375M2 cells. Significance was calculated for protein depleted cell lines compared to wild type cells. Data are mean values +/- S.E.M., over 3 independent experiments; *=P<0.05. Densitometric data was normalised to GAPDH as a loading control.**F.** A-375M2 cells were pre-incubated with 5uM IPA-3 (IPA) for 2 hours and then seeded onto TRITC gelatin coated coverslips for 3 hrs. Coverslips were stained and quantified as described above (A).

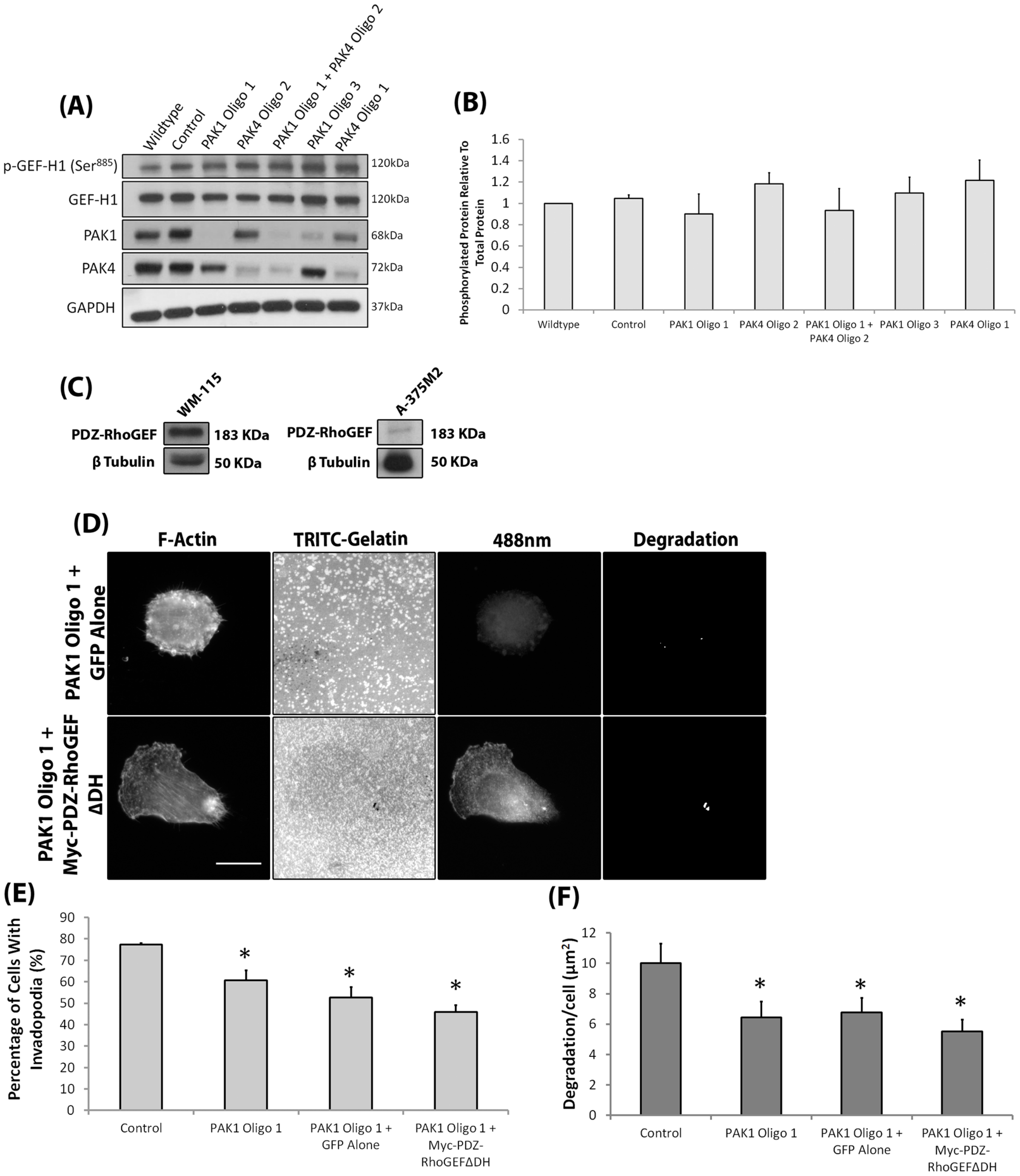

Supplementary Figure S2: PAK1 induced invadopodia function does not signal via PDZ-RhoGEF. A. Western blot of p-GEF-H1(S885), total GEF-H1, PAK1 and PAK4 in WM-115 cells in which PAK1 and PAK4 expression was reduced (individually and simultaneously). **B.** Analysis of western blot data via densitometry showing p-GEF-H1(S885) levels compared to the total GEF-H1 protein. Significance was calculated compared to control WM-115 cells. Data are mean values +/- S.E.M., over 3 independent experiments; *=P<0.05. Densitometric data was normalised to total GEF-H1 levels. GAPDH was used as a loading control. **C.** Western blot of endogenous PDZ-RhoGEF in WM-115 and A375M2 cells. B-tubulin was used as a loading control. **D.** representative images of PAK1 knockdown WM-115 cells expressing control GFP lone or myc-PDZ-RhoGEFΔDH. Scale Bars = 10um. The percentage of cells with invadopodia **E.** and the area of degradation **F.** in PAK1 knockdown cells expressing myc-PDZ-RhoGEFΔDH. Significance was calculated to control WM-115 cells. Data are mean values +/- S.E.M. of 150 (percentage of invadopodia) or 90 (degradation) cells, over 3 independent experiments; *=P<0.05

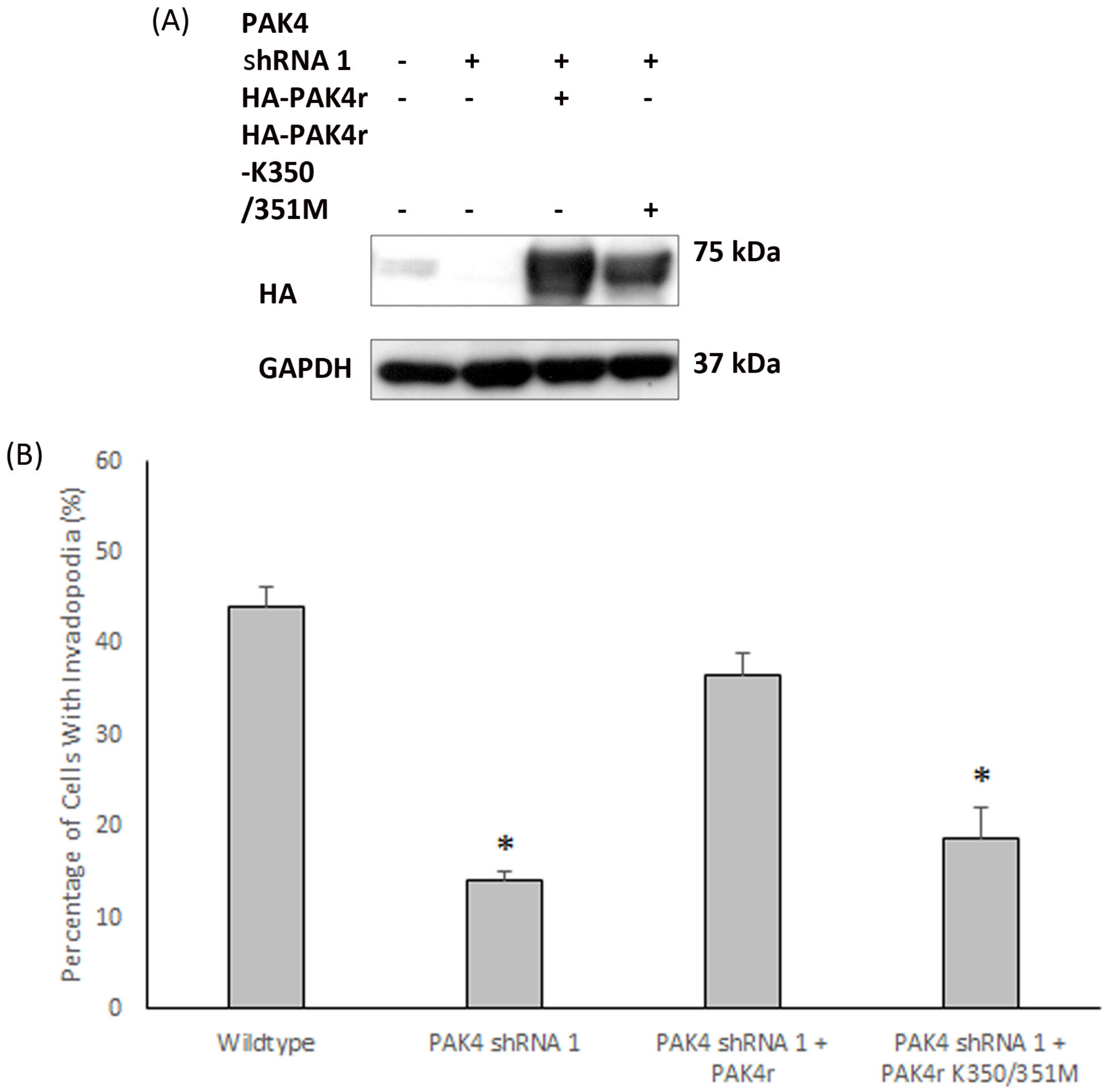

Supplementary Figure S3: Kinase dead PAK4 cannot rescue phenotype. A. Western blot of cell lysates from Wt cells or cells stably expressing PAK4 ShRNA1 which has been transfected with HA-tagged PAK4 shRNA1 resistant construct (PAK4r) or HA-tagged kinase dead PAK4 shRNA1 resistant construct PAK4r-K350/351M. Lysates were probed for HA and GAPDH as a loading control. **B.** The percentage of cells with invadopodia. Significance was calculated to wild type cells. 90 cells, over 3 independent experiments; *=P<0.05
